# National Association of Dental Faculties

**Published:** 1895-12

**Authors:** 


ARTICLE VII.

### NATIONAL ASSOCIATION OF DENTAL FACULTIES.

Courtesy "Dental Cosmos."

The Twelfth Annual Meeting of the National Association of Dental Faculties was held at the Ocean Hotel, Asbury Park, N. J., commencing Saturday, August 2, 1895; the president, Dr. Frank Abbott, in the chair. The entire membership of the association was represented at this meeting as follows:

University of California, Dental Department—L. L. Dunbar.

University of Denver, Dental Department—R. B. Weiser.

Columbian University, Dental Department—J. Hall Lewis.

National University, Dental Department—J. Roland Walton.

Southern Medical College, Dental Department—Frank Holland.

American College of Dental Surgery—Louis Ottofy.

Chicago College of Dental Surgery—Truman W. Brophy.

Northwestern College of Dental Surgery—J. A. Whipple.

Northwestern University Dental School—George H. Cushing.

Indiana Dental College—George Edwin Hunt.

University of Iowa, Dental Department—A. O. Hunt.

Louisville College of Dentistry—Francis Peabody.

Baltimore College of Dental Surgery—M. W. Foster.

University of Maryland, Dental Department—F. J. S. Gorgas.

Boston Dental College—J. A. Follett.

Harvard University, Dental Department—Thomas Fillebrown.

Dental College of the University of Michigan.—J. Taft.

Detroit College of Medicine, Dental Department—G. S. Shattuck.

University of Minnesota, College of Dentistry—Thomas E. Weeks.

Kansas City Dental College—J. D. Patterson.

Western Dental College—D. J. McMillen.

Missouri Dental College—A. H. Fuller.

University of Buffalo, Dental Department—W. C. Barrett.

New York College of Dentistry—Frank Abbott.

Ohio College of Dental Surgery—H. A. Smith.

Western Reserve University, Dental Department—H. L. Ambler.

Pennsylvania College of Dental Surgery—C. N. Peirce.

Philadelphia Dental College—S. H. Guilford.

University of Pennsylvania, Dental Department—James Truman.

Meharry Medical School of Central Tennessee College, Dental Department—G. W. Hubbard.

University of Tennessee, Dental Department—J. P. Gray.

Vanderbilt University, Dental Department—Henry W. Morgan.

Royal College of Dental Surgeons of Ontario—J. B. Willmott.

The following colleges were admitted to membership:

University College of Medicine, Dental Department, Richmond, Virginia—L. M. Cowardin.

Atlanta Dental College—Wm. Crenshaw.

Birmingham Dental College—T. M. Allen.

Cincinnati College of Dental Surgery—G. S. Junkerman.

Cleveland University of Medicine and Surgery, Dental Department—S. B. Dewey.

The following, laid over under the rules from last year, were adopted as here given:

*Resolved,* That in view of the recommendation of the Executive Committee that this Association now in session shall require that all colleges, members of this Association, shall extend the term of the session of 1896-97, and of succeeding sessions, to not less than six months each.

Beginning with the session of 1895-96, no college shall be permitted to retain membership in this Association if it is conducted or managed, in whole or in part, by any person or persons who do not practice dentistry in accordance with well recognized and generally accepted forms, generally known as dental ethics, or if they are owned in whole or in part by men or women who are engaged in disreputable dental practice, or if any college have upon its list of trustees, the faculty, demonstrators, or in any other capacity, any one who does not practice dentistry in accordance with the principles above mentioned. This shall refer to dentists only.

Beginning with the session of 1896-7 the examinations conducted by the colleges of this Association shall be in the English language only.

The other resolutions which came over from last year for action were laid on the table.

A resolution was adopted requiring each college holding membership in the Association to file with the secretary, sixty days before the next meeting, a detailed statement of its equipment and facilities for teaching; all new applicants to file a similar statement with their applications. The secretary was instructed to have blank forms printed for the purpose and forwarded to the various schools.

The report of the special committee on preliminary examinations was received and the committee discharged.

The following resolutions offered by Dr. Patterson were adopted :

*Resolved*, That students in attendance at colleges of this Association are required to obey the laws regulating the practice of dentistry in the various States, and failing to do this, shall not again be received into any of the colleges of this Association.

*Resolved*, That when a college of this Association has increased the cost of tuition fees, no student shall be received at the former fee except those who have matriculated at such college prior to such action.

The Committee on Text-books reported in favor of the adoption as text-books by the colleges of the Association of two works, namely: "Dental Anatomy," by G. V. Black, M. D., D. D. S., and "Methods of Filling Teeth," by Rodrigues Ottolengui, M. D. S. The report was adopted.

The following lie over until next year :

Amendment to the rules offered by the Executive Committee :

That each college be allowed two delegates, and be limited to one vote for each school.

By Dr. Peabody :

That when a student who has matriculated within the time limit in any recognized college shall, from sickness, death or sickness in family, lack of funds, or other reasonable cause, be compelled to retire from that college before the expiration of the term, he may be allowed to make up the deficit of time in the same or any other college (provided he enter at a date not later than that on which he retired), be examined by the last college entered, and if the examination be up to the requirements of that college, and otherwise satisfactory, may be given tickets for advanced standing or graduated, as the case may be.

By Dr. George Edwin Hunt:

Amend the last portion of Rule 3 to read as follows:

"Except on such conditions as would have been imposed in the original school, and these to be ascertained by conference with the school from whence he came."

By Dr. Gray:

Moved that when students from one college apply for advanced standing to any other college of this Association it shall be the duty of the Dean or Secretary of the latter college to ascertain by correspondence with the college from which the student comes if there be any objection to his acception.

By Dr. Gray:

*Resolved*, That all colleges of this Association shall charge not less than one hundred dollars tuition each session.

By Dr. A. O. Hunt:

*Resolved*, That a student who is suspended or expelled from any college of this Association shall not be received by any other college during that current session.

In case the action of the first college is expulsion the student shall not be given credit at any time for the course from which he was expelled.

Any college suspending any student shall at once notify all other members of this Association of its action.

The following resolution offered by Dr. Ottofy was adopted:

*Resolved*. That the endorsement of applications for membership, made during the coming year, shall be based upon definite knowledge obtained by a careful examination of the methods of teaching, the equipment, and the efficiency of the faculty.

The report of the Committee on Revision of the Constitution, Laws and Codified Rules was considered section by section, and laid over for final action next year; and the committee, consisting of Drs. Louis Ottofy, A. O. Hunt and J. D. Patterson, was continued.

The following were elected officers for the ensuing year: S. H. Guilford, president; George H. Cushing, vice-president; Louis Ottofy, secretary; Henry W. Morgan, treasurer; J. Taft, Thomas Fillebrown, B. Holly Smith, executive committee; H. A. Smith, A. O. Hunt and T. W. Brophy, ad interim committee.

The newly elected officers were installed and the president announced the standing committees, as follows: J. A. Follett, L. L. Dunbar, Geo. Edwin Hunt, C. N. Peirce and T. W. Brophy, committee on schools; J. D. Patterson, A. O. Hunt, J. B. Willmott, T. E. Weeks and J. P. Gray, committee on text-books.

Adjourned to meet at the call of the Executive Committee.

